# Chromosome 11q loss and *MYCN* amplification demonstrate synthetic lethality with checkpoint kinase 1 inhibition in neuroblastoma

**DOI:** 10.3389/fonc.2022.929123

**Published:** 2022-09-27

**Authors:** Kaylee M. Keller, Thomas F. Eleveld, Linda Schild, Kim van den Handel, Marlinde van den Boogaard, Vicky Amo-Addae, Selma Eising, Kimberley Ober, Bianca Koopmans, Leendert Looijenga, Godelieve A.M. Tytgat, Bauke Ylstra, Jan J. Molenaar, M. Emmy M. Dolman, Sander R. van Hooff

**Affiliations:** ^1^ Department of Research, Princess Máxima Center for Pediatric Oncology, Utrecht, Netherlands; ^2^ Department of Pathology, Cancer Center Amsterdam, Amsterdam UMC, Vrije Universiteit, Amsterdam, Netherlands; ^3^ Department of Pharmaceutical Sciences, University Utrecht, Utrecht, Netherlands; ^4^ Children’s Cancer Institute, Lowy Cancer Centre, UNSW Sydney, Kensington, NSW, Australia; ^5^ School of Women’s and Children’s Health, Faculty of Medicine, UNSW Sydney, Kensington, NSW, Australia

**Keywords:** pediatric cancer, neuroblastoma, checkpoint kinase 1 (CHK1), chromosome 11q deletion, MYCN amplification, prexasertib, replication stress, synergy

## Abstract

Neuroblastoma is the most common extracranial solid tumor found in children and despite intense multi-modal therapeutic approaches, low overall survival rates of high-risk patients persist. Tumors with heterozygous loss of chromosome 11q and *MYCN* amplification are two genetically distinct subsets of neuroblastoma that are associated with poor patient outcome. Using an isogenic 11q deleted model system and high-throughput drug screening, we identify checkpoint kinase 1 (CHK1) as a potential therapeutic target for 11q deleted neuroblastoma. Further investigation reveals *MYCN* amplification as a possible additional biomarker for CHK1 inhibition, independent of 11q loss. Overall, our study highlights the potential power of studying chromosomal aberrations to guide preclinical development of novel drug targets and combinations. Additionally, our study builds on the growing evidence that DNA damage repair and replication stress response pathways offer therapeutic vulnerabilities for the treatment of neuroblastoma.

## Introduction

Neuroblastoma (NB) is a malignancy of the sympathetic nervous system and is the most common extracranial solid tumor found in children (1). Based on clinical and molecular features, such as *MYCN* amplification, NB can be classified as low-, intermediate- or high-risk (2). Contrasting with the standard of care procedure for low-risk NB, which includes observation and sometimes surgery, high-risk patients undergo intensive chemotherapy, surgery, radiation therapy and immunotherapy (3). While most high-risk NB tumors initially respond to treatment, relapse and therapy resistance remain major clinical obstacles. Approximately 50% of high-risk patients eventually succumb to the disease, thus there is an absolute need for more effective therapeutic approaches for these patients (1).

Over the last decades, intense efforts have been made to develop targeted therapies for NB patients; however, breakthroughs have been hindered by the paucity of recurrent somatic mutations. Activating mutations of the ALK tyrosine kinase receptor remain the only targetable recurrent somatic variant observed in NB at diagnosis (4). Although targeted inhibition of ALK is a promising approach for ALK mutated NB specifically, only 8-10% of tumors harbor this aberration, thereby limiting the practical application of these inhibitors (5).

Rather than a mutationally driven (M class) landscape, a remarkable number of NB tumors are driven by chromosomal aberrations, which groups them with the copy number driven (C class) tumor entities (6). Hemizygous loss of chromosome 11q is observed in approximately 35-45% of all NB tumors and represents a subgroup of patients with a poor prognosis (7–10). Using whole genome sequencing (WGS) and single-nucleotide polymorphism (SNP) analysis, recent studies have investigated the impact that 11q loss has on NB and suggest that 11q deletion leads to an undifferentiated cell state by altering the expression of candidate tumor suppressor genes *DLG2* and *SHANK2* (11, 12). However, due to the difficulty in modeling large-scale chromosomal copy number aberrations using conventional molecular biology techniques, the effects of these structural variants and the potential therapeutic vulnerabilities mediated by them remain relatively unexplored.

In this study, we used an isogenic 11q deleted NB model system together with high-throughput drug screening to uncover checkpoint kinase 1 (CHK1) as a potential drug target for 11q deleted NB. Further investigation revealed *MYCN* amplification (MNA) as a potential additional biomarker for CHK1 inhibition and high-throughput combination drug screens identify WEE1 kinase (WEE1) inhibition as a synergistic candidate across all 11q and *MYCN* phenotypes. Altogether our study demonstrates the potential of using chromosomal aberrations to guide preclinical development of targeted therapeutic approaches and adds to the mounting evidence that CHK1 might be an effective therapeutic target for the treatment of NB (13).

## Methods

### Cell lines

The cell lines IMR-32, NGP, SJNB-6, CHLA-90, SKNAS, Gimen, SKNBE, SJNB-8, KCNR, SJNB-12, Shep2, SH-SY5Y and SKNSH were obtained *via* the American Type Culture Collection (ATCC) and Shep21n cells were obtained *via* historic collaboration. The identity of all cell lines was validated by short tandem repeat (STR) analysis and phenotypic observation. For all cell lines except for CHLA-90, cells were cultured in Dulbecco’s Modified Eagle Medium (DMEM; Thermo Fisher Scientific, #41965) supplemented with 10% (v/v) fetal bovine serum, 2 mM L-glutamine, 1% (v/v) non-essential amino acids and 100 U/mL penicillin and 100 mg/mL streptomycin. CHLA-90 was cultured in Iscove’s Modified Dulbecco’s Medium (IMDM; Thermo Fisher Scientific, #12440) supplemented with 1% (v/v) insulin-transferrin-selenium (Thermo Fisher Scientific, #41400), 10% (v/v) fetal bovine serum, 2 mM L-glutamine and 100 U/mL penicillin and 100 mg/mL streptomycin.

Patient-derived neuroblastoma tumoroid NB139 was grown in DMEM-GlutaMAX (Thermo Fisher Scientific, #21885) supplemented with 25% (v/v) Ham’s F-12 nutrient mixture, B27 supplement minus vitamin A, 100 U/mL penicillin, 100 mg/mL streptomycin, 20 ng/mL epidermal growth factor (EGF) and 40 ng/mL fibroblast growth factor-basic (FGF-2). AMC772, NB129 and NB059 were grown in DMEM-GlutaMAX (Thermo Fisher Scientific, #21885) supplemented with 20% (v/v) Ham’s F-12 nutrient mixture, B27 supplement minus vitamin A, N-2 supplement, 100 U/mL penicillin, 100 mg/mL streptomycin, 20 ng/mL epidermal growth factor (EGF), 40 ng/mL fibroblast growth factor-basic (FGF-2), 200 ng/mL insulin-like growth factor-1 (IGF-1), 10 ng/mL platelet-derived growth factor-AA (PDGF-AA) and 10 ng/mL platelet-derived growth factor-BB (PDGF-BB). Additionally, NB059 was supplemented with 10% (v/v) human plasma (Thermo Fisher Scientific, P9523). EGF, FGF-2, PDGF-AA and PDGF-BB were obtained from PeproTech, IGF-1 was obtained from R&D Systems, and B27 minus and N-2 supplement were obtained from Thermo Fisher Scientific.

Chromosome 11q status of all *in vitro* models was verified *via* whole genome sequencing (WGS; NovaSeq 6000; https://www.ebi.ac.uk/ena/browser/view/PRJEB54725).

### SKNSH clones

SKNSH 11q deleted clones (clones 4, 7 and 10) and SKNSH 11q wild type clones (clones 1, 8 and 11) were generated as previously described (14). In short, deletion of 11q was induced using CRISPR-Cas9 and single clones were selected and grown out to establish SKNSH cell lines with and without 11q loss (14). Cell lines were maintained in Dulbecco’s Modified Eagle Medium (DMEM; Thermo Fisher Scientific, #41965) supplemented with 10% (v/v) fetal bovine serum, 2 mM L-glutamine, 1% (v/v) non-essential amino acids and 100 U/mL penicillin and 100 mg/mL streptomycin. The chromosome 11q status of these *in vitro* models were verified *via* PCR (primers provided in **Supplementary Table 1**).

### Compound screening

Using the Multi-drop™ Combi Reagent Dispenser (Thermo scientific), classical cell lines and patient-derived tumoroids were seeded in duplicate in black 384-well plates (Corning, 3764) at a density of 400-20000 cells per well depending on the line being used. Following a 24h-period given to allow cells to attach, cells were treated with compounds.

For high-throughput screens, screening experiments and processing were performed by the high-throughput screening facility of the Princess Máxima Center (https://research.prinsesmaximacentrum.nl/en/core-facilities/high-throughput-screening). The Echo550 dispenser was used to add a library of 197 drugs in dose ranges of six concentrations between 0.1 nM and 10 µM, with a final DMSO concentration of 0.25% (**Supplementary Table 2**). Several drugs were tested at additional lower concentrations (up to 10 pM) or higher concentrations (up to 200 µM).

For monotherapy and combination validation, 0.03-10 µM of prexasertib (Cat: HY-18174), adavosertib (Cat: HY-10993), SN-38 (Cat: HY-13704) and/or topotecan (Cat: HY-13768A) were added using the D300e Digital Dispenser (TECAN). After 72 hours of drug treatment at normal culture conditions, cell viability of classical lines was measured using the 3-(4,5-dimethylthiazol-2yl)-2,5-diphenyltetrazolium (MTT) assay and patient-derived tumoroid viability was measured using CellTiter-Glo 3D^®^ (Promega) according to the manufacturer’s instructions (15).

### Western blot analysis

Following protein harvest using Laemmli lysis buffer, protein concentrations of whole cell extracts were measured using DC protein Assay (Bio-Rad). Next, equal amounts of protein were loaded onto a Bio-Rad Mini-Protean^®^ TGX™ 4-20% gel. Proteins were subsequently transferred onto polyvinylidene difluoride (PVDF) membranes, after which membranes were blocked using ECL advance blocking agent (GE Healthcare) in TBS-Tween 0.1%. Proteins of interest were detected using the following antibodies: anti-N-myc (Cell Signaling, Cat: 9405), anti-CHK1 (Cell Signaling, Cat: 2360), anti-phospho-CHK1 S296 (Cell Signaling, Cat: 2349), anti-WEE1 (Cell Signaling, Cat: 13084), anti-CDC2 (Cell Signaling, Cat: 9116), anti-phospho-CDC2 Y15 (Cell Signaling, Cat: 4539), anti-γH2AX (Abcam, Cat: ab26350), anti-alpha-tubulin (Cell Signaling, Cat: 3873), anti-beta-actin (Cell Signaling, Cat: 4967). Following treatment with HRP-link secondary antibodies (Invitrogen), detection was performed using Bio-Rad Chemidoc™ Touch (BioRad).

### Cell cycle analysis

Cells were treated with 3.2 or 5 nM prexasertib and/or 16 nM adavosertib for 0-72 hours. Next, floating and adherent cells were harvested, washed with PBS and resuspended in PBS with 2-4 mM EDTA. Prior to FACS measurement, cells were either 1) fixed with 100% ethanol and then resuspended in PBS with 0.1 mg/ml propidium iodide and 1 mg/mL RNAse for 45 minutes or 2) stained with Vybrant Dyecycle violet (1:1000, Thermo Fisher) for 30 minutes. Lastly 20000 cells were measured *via* flow cytometry and data was analyzed using FlowJo software (FlowJo) and CytExpert software (Beckman).

### Benchwork dataset and statistical analysis

Drug sensitivity data generated using CellTiter-Glo (Promega) cell viability assay (GDSC2) was downloaded from the Genomics of Drug Sensitivity in Cancer website (16). The 11q status of the included neuroblastoma cell lines was screened using copy number variation data obtained from the Sanger Institute Catalogue of Somatic Mutations in Cancer website, http://cancer.sanger.ac.uk/cosmic (17). Students T-test were performed to determine the significance of differences in IC_50_ between 11q wild-type and 11q deleted neuroblastoma cell lines. The Mann-Whitney U test was used to make comparisons between the average IC_50_ of two groups. For all statistical analyses, a p-value > 0.05 was deemed non-significant and p ≤ 0.05 (*), p ≤ 0.01 (**), and p ≤ 0.001 (***) were considered to be statistically significant.

## Results

### Chromosome 11q loss induces sensitivity to CHK1 inhibition

**Figure 1:**
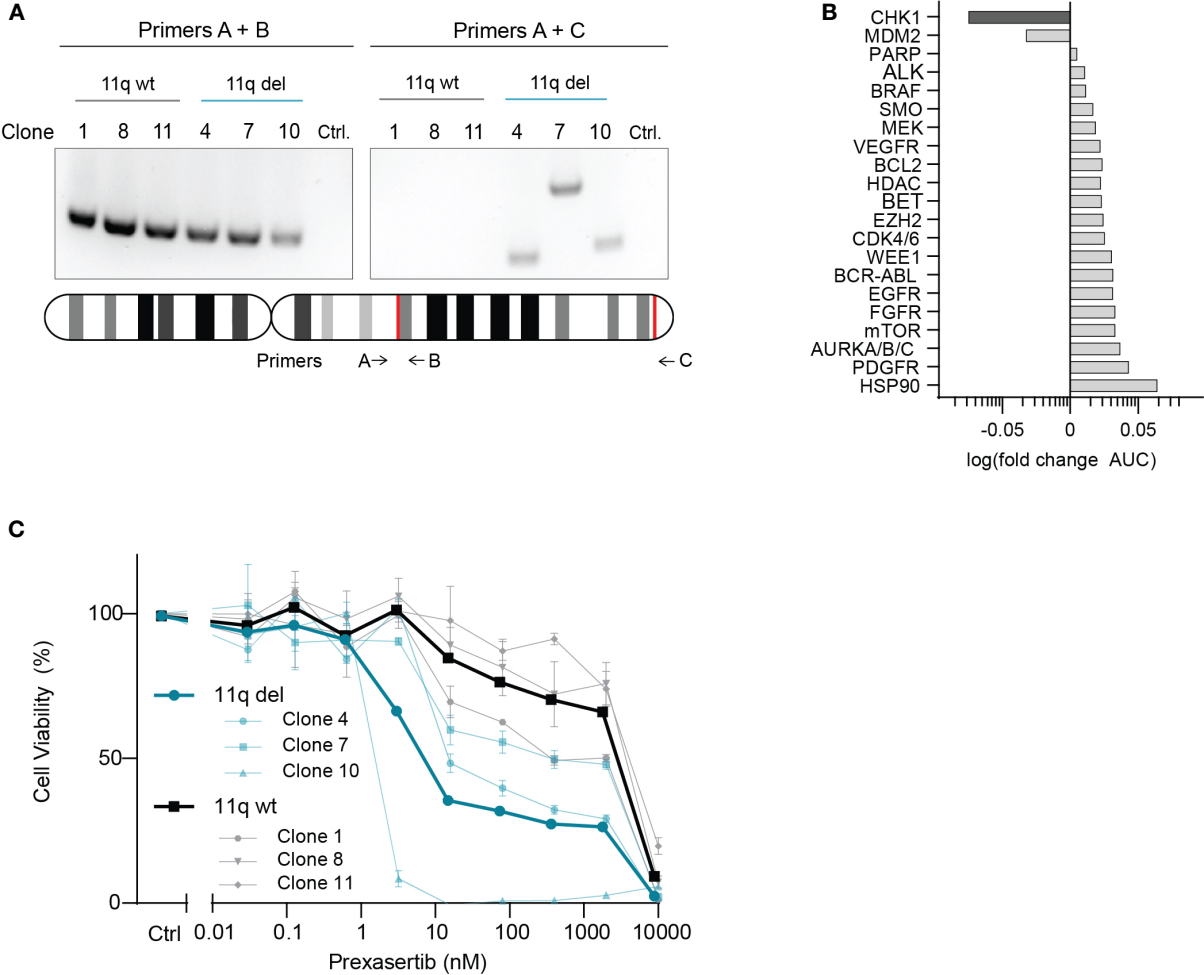
CHK1 identified as a therapeutic target in 11q deleted NB. **(A)** Agarose gel electrophoresis of PCR products from 11q wild type (black) and 11q deleted (blue) SKNSH clones using primer set A + B (left) to amplify the wild type copy of 11q, and primer set A + C (right) which only yields a product if 11q deletion was induced. Shown below is an ideogram of chromosome 11q with the approximate location of primer targets and the gRNA recognition sites for CRISPR-Cas9 directed 11q deletion (red). **(B)** Median log fold change in area under the curve (AUC) for 11q deleted and wild type SKNSH clones following 72-hour incubation with compounds targeting the proteins listed on the y-axis. **(C)** Dose-response curves for three SKNSH clones with chromosome 11q deletion (Clone 4, 7, 10; blue) and three SKNSH clones with a normal chromosome 11q locus (Clone 1, 8, 11; grey). Average dose-response curves for each phenotype are indicated in bold. All curves represent the average of replicates (n=2), and error bars indicate the standard error of the mean (SEM).

To investigate potential drug vulnerabilities mediated by chromosome 11q deletion, we used an isogenic model system that was developed using the 11q wild type NB cell line, SKNSH (14). CRISPR-Cas9 was used to induce a large chromosome 11q deletion, recapitulating the most frequently observed 11q loss in NB (ch11q13.4-25) and single cells were isolated and grown to establish the monoclonal SKNSH lines used in our study (14). Following confirmation of heterozygous loss of 11q using two independent PCR primer sets (**Figure 1A**), three SKNSH clones with wild type 11q and three SKNSH clones with proven 11q deletion were exposed to a drug library containing 197 compounds that are approved or in (pre)clinical development for pediatric cancer. To select compounds that were overall more effective in clones with 11q loss, we calculated the average log fold change in the area under the curve (AUC) between 11q deleted and 11q wild type clones for each compound. Of the entire compound library, CHK1 inhibition with prexasertib resulted in the largest negative fold change in AUC, indicating improved efficacy in 11q deleted SKNSH clones (**Supplementary Figure 1**). Considering our goal of identifying novel therapeutics for the treatment of NB, we selected hits to include only compounds that are currently being clinically investigated in NB (**Supplementary Table 3**). Of the targets currently in clinical trials for NB, CHK1 and MDM2 were the only two targets that demonstrated improved sensitivity in the presence of 11q loss (**Figure 1B**). Since MDM2 inhibitors have been extensively studied *in vitro* in NB (18–20), we proceeded with validation of our top hit of targeted inhibition of CHK1 with prexasertib (LY2606368). Following testing with a wider range of concentrations on the generated SKNSH cell lines, we observed that clones harboring an 11q deletion had IC_50_ values that were on average nearly 300 times lower (average IC_50_ = 9.1 nM) compared to that of SKNSH clones with a wild type 11q locus (average IC_50_ = 2508.4 nM; **Figure 1C**). As SKNSH is a heterogenous cell line, it is likely that the expanded subclones represent different phenotypes of the SKNSH cell line, which could potentially influence sensitivity to CHK1 inhibition (21, 22).

**Figure 2:**
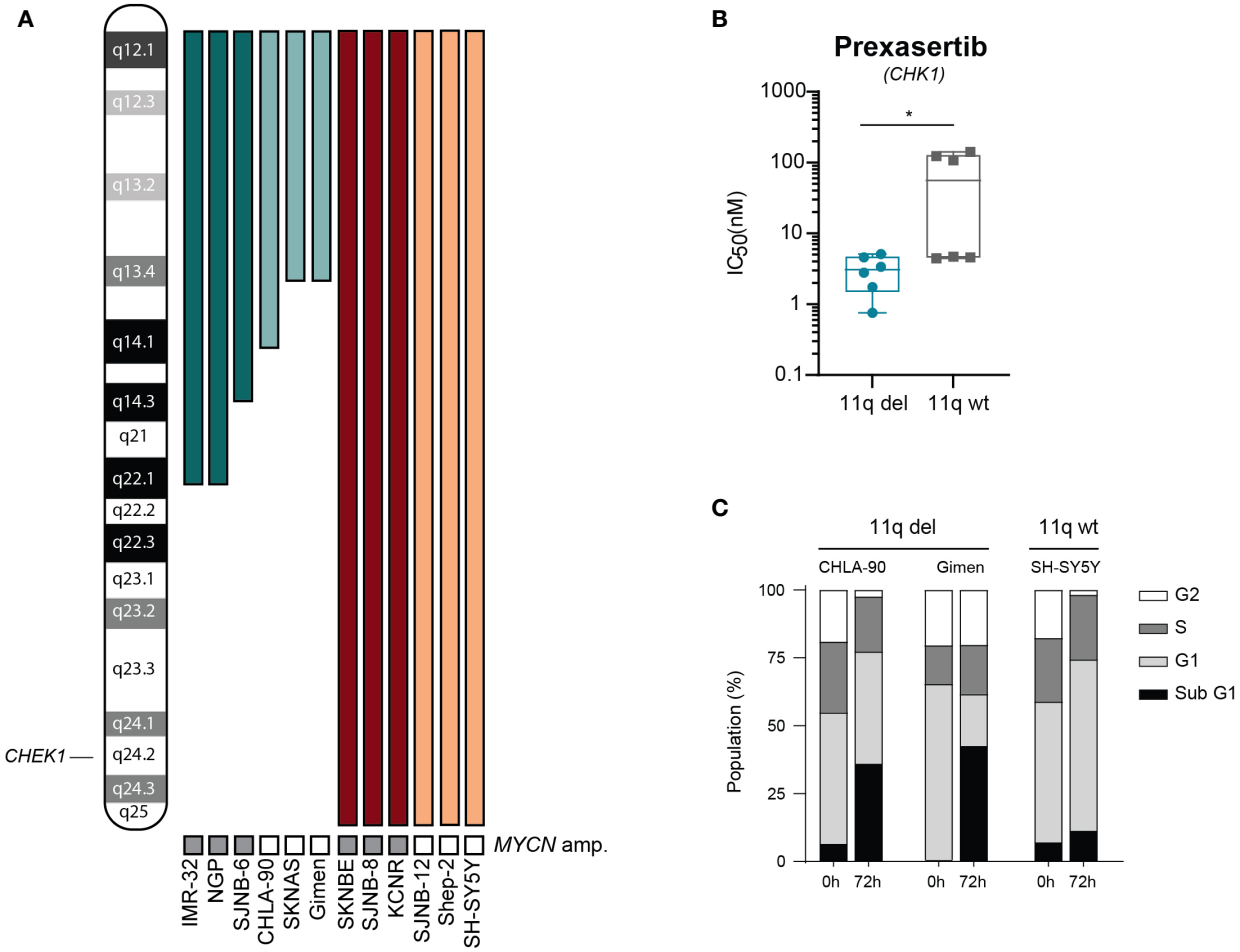
Cells with 11q loss are more sensitive to prexasertib treatment. **(A)** Ideogram of chromosome 11q and approximate deletions (represented by the absence of a colored bar) in the panel of NB cell lines used in this study. **(B)** Box plot of IC_50_ values following 72-hour prexasertib treatment in 11q del (blue) and 11q wild type (grey) cell lines. Statistical significance is reached (Mann Whitney U test, p=0.03). **(C)** Cell cycle distribution of 11q deleted (CHLA-90 and Gimen) and 11q wild type (SH-SY5Y) cells following 72-hour incubation with 5 nM of prexasertib.

To further explore differences in sensitivity to CHK1 inhibition and to validate our observation that 11q deletion is correlated with sensitivity to CHK1 inhibition, we tested prexasertib in a panel of 12 NB cell lines (outlined in **Figure 2A**) that were selected based on the presence or absence of 11q loss as determined by WGS. Following prexasertib treatment, we observed that cell lines with an aberrant 11q locus had a significantly lower average IC_50_ value (3.1 nM) compared to the average IC_50_ of 11q wild type cell lines (64.2 nM; **Figure 2B** **;** **Supplementary Table 4**). Further validation using propidium iodide staining and cell cycle analysis was consistent with these observations: cell lines with an 11q deletion had a greater proportion of sub G1 cells (>25%) compared to 11q wild type cells (10%; **Figure 2C**).

**Figure 3:**
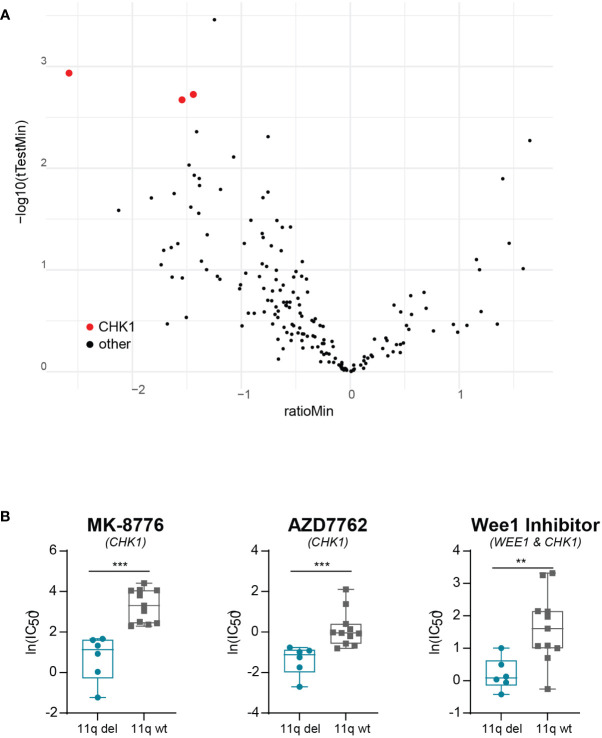
Validation of correlation between 11q loss and CHK1 inhibitor sensitivity. **(A)** Volcano plot of differential drug sensitivities in 11q deleted versus 11q wild type neuroblastoma cell lines included in the Wellcome Trust Sander Institute dataset. The y-axis shows the inverted p-value (-log10) as derived by student’s t-test and the x-axis shows the effect magnitude (mean IC50 of 11q deleted – mean IC50 of 11q wild type cell lines). Each dot represents one compound and those targeting CHK1 are highlighted in red. **(B)** Box plot of IC50 values of NB cell lines included in the Wellcome Trust Sanger Institute dataset after exposure to CHK1 inhibition with MK-8776 (p = 0.0002), AZD7762 (p = 0.0003) or Wee1 Inhibitor (p = 0.0071). Statistical significance is reached (Mann-Whitney U test).

To exclude the possibility that our observations were due to off-target effects specific to prexasertib, we made use of the publicly available dataset from the Wellcome Trust Sanger institute, which includes two different CHK1 inhibitors (MK-8776 and AZD7762). Using this dataset, we observed that NB cell lines with 11q loss were significantly more sensitive to CHK1 inhibition than 11q wild type cells (**Figures 3A, B**). Additionally, the dataset included a WEE1 inhibitor which also targets CHK1 (Wee1 Inhibitor). Again, 11q deleted cell lines demonstrated superior sensitivity to this compound, despite the fact that our results presented in **Figure 1B** suggest that 11q deleted cells are less sensitive to specific WEE1 inhibition (using adavosertib). Altogether, our observations indicate that the improved efficacy associated with 11q deleted cells is likely an effect related to specific targeting of CHK1.

### *MYCN* amplification induces sensitivity to prexasertib in 11q wild type cells

**Figure 4:**
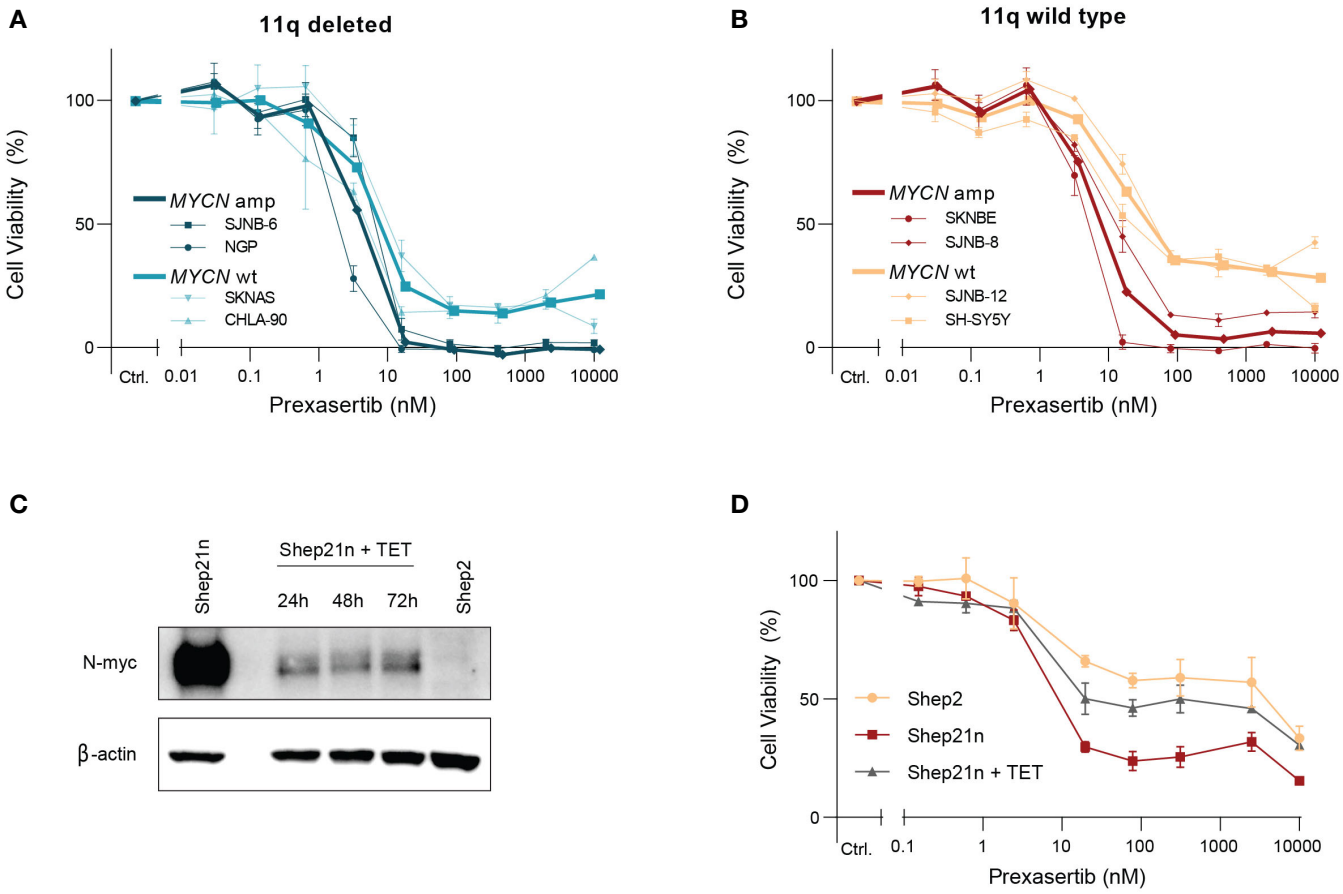
*MYCN* amplification induces sensitivity to prexasertib treatment in 11q wild type cells. **(A)** Dose-response curves of two 11q deleted cell lines with MNA (dark blue; NGP and SJNB-6) and two *MYCN* wild type cell lines (light blue; CHLA-90 and SKNAS) following 72-hour treatment with prexasertib. **(B)** Dose-response curves of two 11q wild type cell lines with MNA (red; SKNBE and SJNB-8) and two *MYCN* wild type cell lines (orange; SH-SY5Y and SJNB-12) following 72-hour treatment with prexasertib. Average dose-response curves are indicated in bold, and all curves represent the average of replicates (n=2) where error bars indicate the standard error of the mean (SEM). **(C)** Western blot of N-myc expression for Shep21n and Shep2 cells without tetracycline treatment and for Shep21n with 50 ng/ml tetracycline treatment for 24, 48 and 72 hours. **(D)** Dose-response curves for 72-hour prexasertib treatment in the cell lines Shep2 (orange), Shep21n (red) and Shep21n with 50 ng/ml tetracycline (grey).

Intriguingly, we observed two clearly distinct groups in the 11q wild type cell lines after exposure to prexasertib—one which is insensitive to prexasertib treatment and one which has IC_50_ values comparable to 11q deleted cell lines (**Figure 2B**). This response dichotomy suggests the presence of other biomarkers and prompted us to consider other genetic abnormalities frequently observed in NB. MNA is one of the most common aberrations, observed in approximately 25% of NB tumors, and is associated with a poor prognosis (23–25). MNA has been shown to increase replication stress and render cells more vulnerable to targeted inhibition of proteins involved in replication stress response pathways, one of which is CHK1 (26, 27). Closer examination of the dose response curves revealed that *MYCN* status had no substantial additional effect on prexasertib sensitivity in 11q deleted cell lines (**Figure 4A**), but that MNA was associated with prexasertib sensitivity in 11q wild type cells (**Figure 4B**).

To further explore this involvement of MNA in prexasertib sensitivity, we used an isogenic model of the Shep21n cell line (28). This is a cell line derived from the *MYCN* and 11q wild type Shep2 cell line with induced constitutive *MYCN* expression that can be downregulated with tetracycline treatment (**Figure 4C**). Using this model system, we indeed observed that constitutive expression of *MYCN* induced prexasertib sensitivity in Shep21n cells (IC_50_ = 7.3 nM versus 579.1 nM in Shep2 cells) and that this effect could be abrogated with tetracycline treatment (IC_50_ = 22.1 nM; **Figure 4D**). These results strongly suggest that MNA could be a biomarker for CHK1 inhibition in 11q wild type tumors and warrants further investigation.

### Combined CHK1 and WEE1 inhibition is synergistic in all 11q and *MYCN* phenotypes

**Figure 5:**
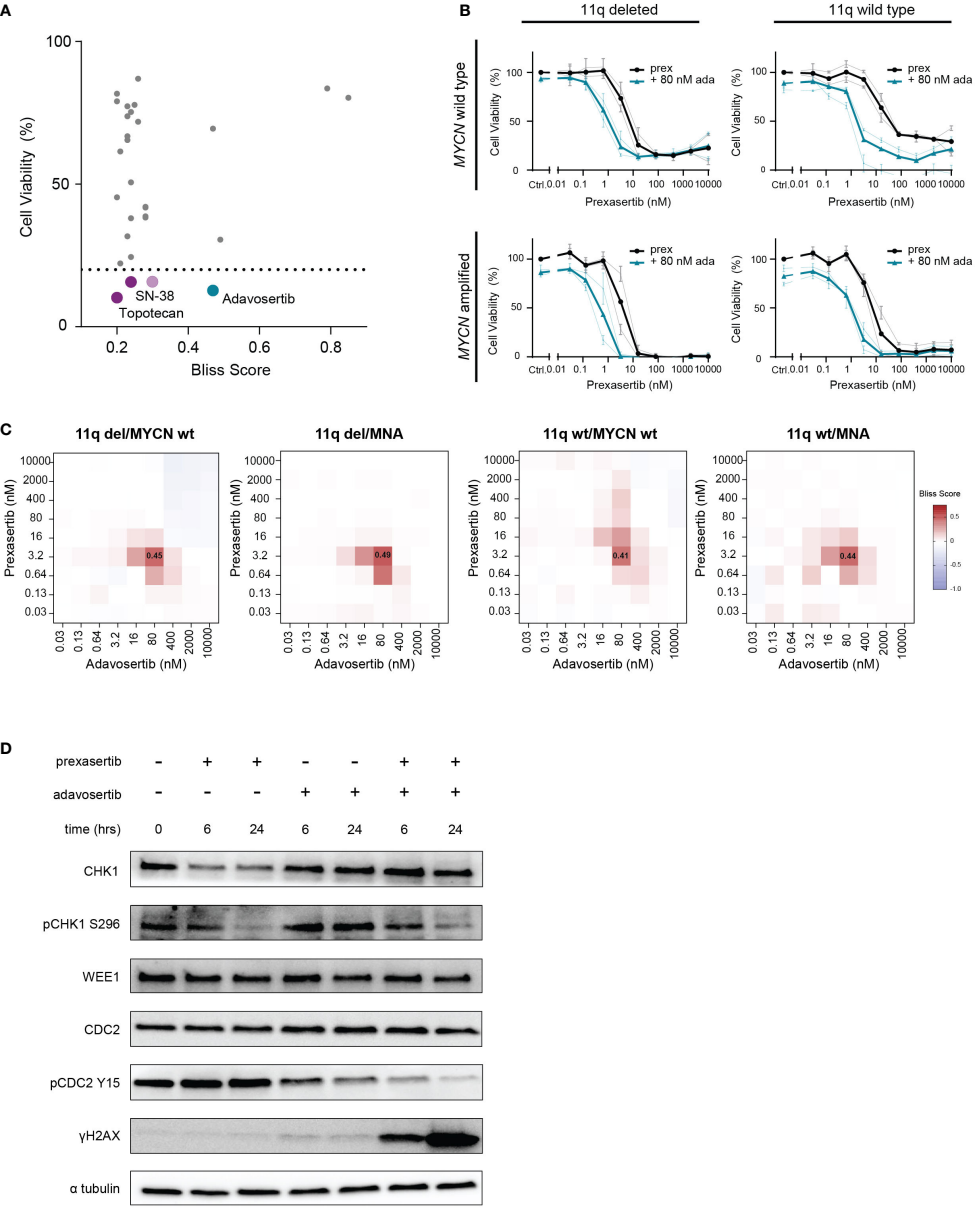
Combined CHK1 and WEE1 inhibition is strongly synergistic. **(A)** High-throughput screening hits that resulted in a bliss independence score > 0.2 when combined with prexasertib treatment for 72 hours in the 11q deleted SKNSH clone 10 cells. Compounds that induced > 80% cell killing are represented in purple (cytotoxic compounds) and blue (targeted inhibitors). **(B)** Dose-response curves for 11q del/*MYCN* wild type (SKNAS and CHLA-90), 11q del/MNA (NGP and SJNB-6), 11q wild type/*MYCN* wild type (SJNB-12 and SH-SY5Y) and 11q wild type/MNA (SJNB-8 and SKNBE) cell lines following 72-hour prexasertib treatment (black) or in combination with 80 nM adavosertib (blue). Average dose-response curves are indicated in bold, and all curves represent the average of replicates (n=2) where error bars indicate the standard error of the mean (SEM). **(C)** Heatmaps indicating the average bliss independence scores for 11q del/*MYCN* wild type (SKNAS and CHLA-90), 11q del/MNA (NGP and SJNB-6), 11q wild type/*MYCN* wild type (SJNB-12 and SH-SY5Y) and 11q wild type/MNA (SJNB-8 and SKNBE) cell lines following combination treatment with 0.03-10 µM prexasertib and adavosertib. **(D)** Protein expression of CHK1, pCHK1 (S296), WEE1, CDC2, pCDC2 (Y15) and γH2AX in SJNB6 cells following 6- or 24-hour treatment with 0.64 nM prexasertib and/or 80 nM adavosertib.

Since monotherapy often leads to the emergence of resistance, we next focused on identifying potential combination strategies. To identify compounds that are synergistic with prexasertib in an 11q deleted phenotype, we performed a high-throughput drug screen, combining prexasertib treatment with a library of 197 cytotoxic and targeted compounds using the prexasertib sensitive, 11q deleted SKNSH clone 10 cells. Following combination treatment, cell viability was calculated and synergy was evaluated using the bliss independence model (29). In our experiment, 29 compounds had a bliss independence score greater than 0.2 when combined with prexasertib, indicating synergism (**Supplementary Table 5**). As synergy alone does not always translate to efficacy, we use cell viability (final cell viability <20%) as an extra selection criterium to identify combinations with relevant therapeutic effects. Using this strategy, we found that topotecan, SN-38 (the active metabolite of irinotecan), cytarabine and adavosertib demonstrated synergism and efficacy when combined with prexasertib (**Figure 5A**).

The strongest hit in our high-throughput drug combination screen, combining synergy and efficacy, was the WEE1 inhibitor, adavosertib (bliss score = 0.47; final cell viability = 12.7%; **Figure 5A**). To validate this observation, we screened this combination using a larger concentration range across a wider panel of NB cell lines representing different 11q and *MYCN* phenotypes (11q del/*MYCN* wt: SKNAS, CHLA-90; 11q del/MNA: NGP, SJNB-6; 11q wt/*MYCN* wt: SJNB-12, SH-SY5Y; 11q wt/MNA: SJNB-8, SKNBE). Consistent with our high-throughput data, we observed strong synergism (bliss score = 0.45) and efficacy when prexasertib was combined with 80 nM of adavosertib to treat 11q deleted, *MYCN* wild type cell lines. Interestingly, this combination also resulted in strong synergy (bliss score = 0.41-0.49) in all other cell lines, regardless of 11q or *MYCN* status, suggesting a general applicability of this combination in NB (**Figures 5B, C**; **Supplementary Figures 2-3**). To investigate the on-target effects of prexasertib and/or adavosertib treatment, we interrogated the phosphorylation status of CHK1 and the downstream target of WEE1, CDC2. Following 6- or 24-hour treatment with prexasertib or adavosertib monotherapy, we observed a decrease in pCHK1 (S296) and pCDC2 (Y15), respectively, and very minor induction of DNA damage as evidenced by γH2AX staining (**Figure 5D**). Following combination therapy with prexasertib and adavosertib, phosphorylation of CDC2 is nearly entirely inhibited and a large accumulation of DNA damage is observed.

**Figure 6:**
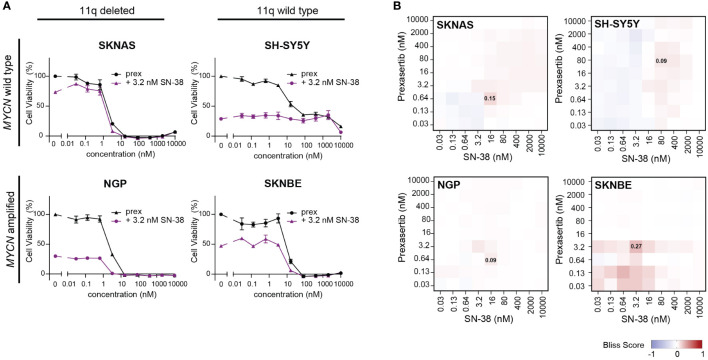
CHK1 inhibition combined with SN-38 treatment demonstrates additive effects. **(A)** Dose-response curves for classical for 11q deleted/wild type cell lines with and without MNA following 72-hour exposure to prexasertib alone (prex, black) or in combination with 3.2 nM of SN-38 (purple). All curves represent the average of replicates (n=2) where error bars indicate the standard error of the mean (SEM). **(B)** Heatmaps indicating the calculated bliss independence scores for cell lines treated with 0.03-10 µM prexasertib and SN-38.

Being that irinotecan and topotecan are standard chemotherapy modalities used in the clinic for the treatment of recurrent or refractory NB, we specifically further explored these hits in SKNAS (11q del/*MYCN* wt), NGP (11q del/MNA), SH-SY5Y (11q wt/*MYCN* wt) and SKNBE (11q wt/MNA) cells. When prexasertib is combined with SN-38 or topotecan, additive/minor synergistic effects are observed across all 11q and *MYCN* phenotypes and no relevant antagonism is measured (**Figures 6A, B**; **Supplementary Figures 4-6**). To further evaluate how CHK1 inhibition interacts with other standard-of-care compounds used in the treatment of NB, more robust preclinical studies should be performed. Nonetheless, the observed additive effects in all NB phenotypes in our study suggest that the addition of CHK1 inhibition could potentially be used to lower the required dose of classic chemotherapeutics and thereby limit the toxic side effects that are often associated with these therapies.

### CHK1 inhibition is effective in patient-derived neuroblastoma model systems and demonstrates synergy when combined with WEE1 inhibition

**Figure 7:**
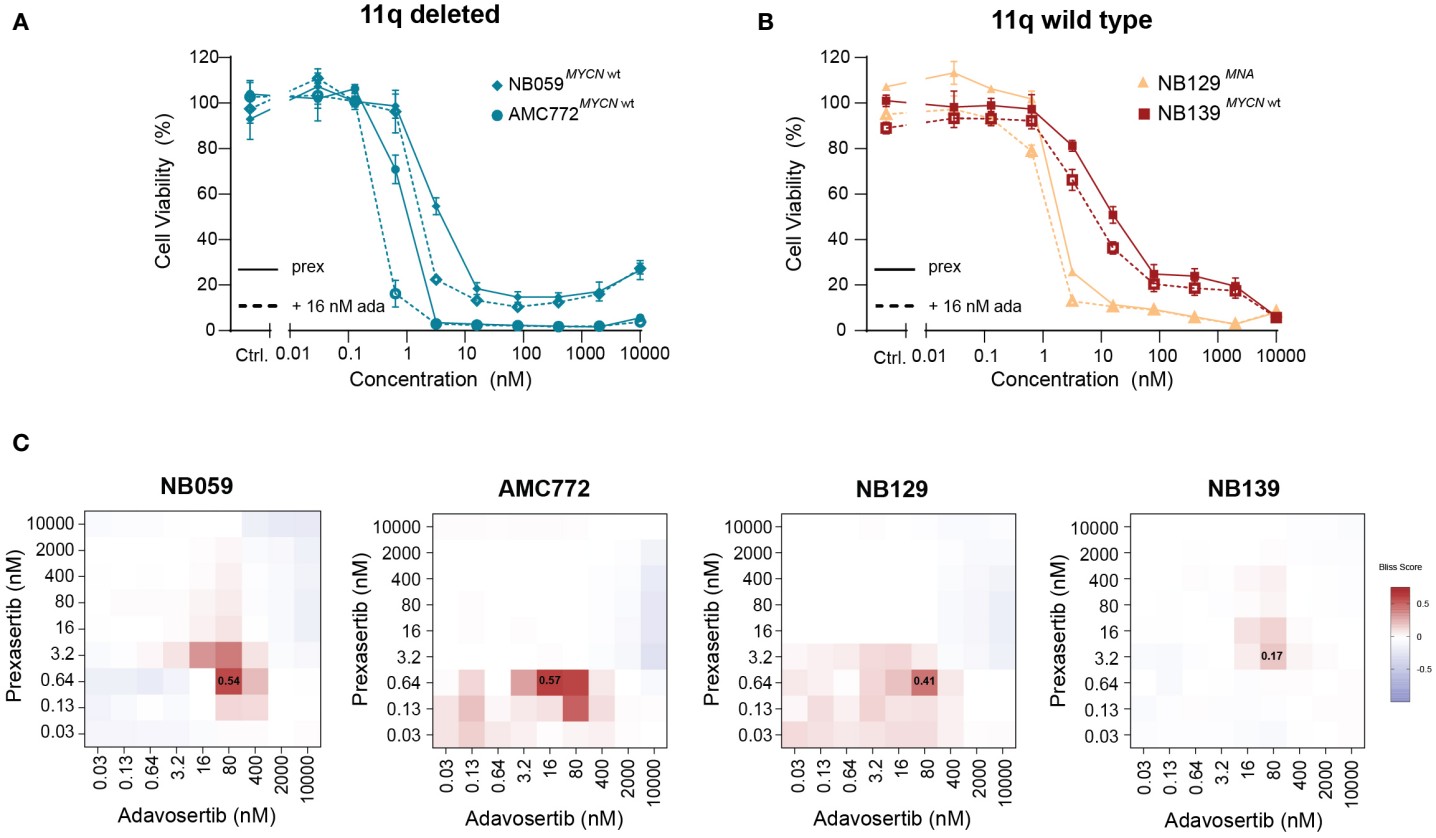
Patient-derived NB tumoroids are sensitive to CHK1 inhibition and improved *in vitro* efficacy is observed with the addition of WEE1 inhibition. **(A)** Dose-response curves of 11q deleted, MYCN wild type NB tumoroids (NB059 and AMC772) following 72-hour treatment with prexasertib only (prex, solid line) or in combination with 16 nM adavosertib (ada, dashed line). **(B)** Dose-response curves of 11q wild type NB tumoroids with MYCN amplification (NB129, orange) and without MYCN amplification (NB139, red) following prexasertib treatment (prex, solid line) or prexasertib in combination with 16 nM of adavosertib (ada, dashed line). All curves represent the average of replicates (n=2) where error bars indicate the standard error of the mean (SEM). **(C)** Bliss independence heatmaps for NB tumoroids following combination treatment with 0.03-10 µM prexasertib and adavosertib.

Next, we used our generated patient-derived NB tumoroids to explore our findings further. Tumoroids more closely recapitulate the genomic background and phenotype of NB tumors than classical cell lines and provide additional *in vitro* evidence to support further studies using combined CHK1 and WEE1 inhibition in NB (30). Following treatment with prexasertib, we observed the same effects as in the classical NB cell lines—tumoroids with an 11q deletion and/or MNA were more sensitive to prexasertib treatment than lines that harbor neither aberration (**Figures 7A, B** **;** **Supplementary Figure 7**). Additionally, we observed synergism in all tumoroids when treated with combined CHK1 and WEE1 inhibitors (**Figure 7C**). The synergistic effects were further validated by evaluating phenotypic and cell cycle changes in tumoroids. Consistent with our previous observations, combination treatment with prexasertib and adavosertib induced greater cell killing compared to monotherapy (**Supplementary Figure 8**).

## Discussion

In our study, we use the characteristic hemizygous loss of chromosome 11q in NB to explore targetable vulnerabilities that can be used for therapeutic purposes. Being a copy number driven (C class) tumor, the development and practical application of targeted inhibitors for the treatment of NB has been challenging as there are few targetable recurrent somatic mutations in these tumors at diagnosis (6). By using specific large-scale chromosomal aberrations to guide the selection and validation of potential drug targets, we hope to expand the clinical applicability of novel targeted therapies and help develop better treatment options for children with NB.

Following high-throughput screening of a drug library including targeted inhibitors that are clinically approved or in (pre)clinical development, we identify CHK1 as a potential target for 11q deleted NB. Interestingly, this is not the first time that CHK1 has been proposed for the treatment of NB. Using RNAi loss-of-function screens, CHK1 was identified as a potent target in NB and further preclinical validation studies have demonstrated exceptional *in vitro* and *in vivo* efficacy of the CHK1 inhibitor, prexasertib (31–33). While there is an ongoing phase I clinical trial for prexasertib in pediatric patients (NCT02808650; **Supplementary Table 3**), including those with NB, a clear biomarker for CHK1 inhibition has yet to be identified. Until now no associations have been made between copy number aberrations and CHK1 inhibition. By further investigating drug sensitivity in cell lines, patient-derived tumoroids and a publicly available dataset—which altogether encompass many different sizes of 11q loss—our study clearly demonstrates that hemizygous deletion of 11q can be used as a biomarker for single compound treatment with prexasertib.

Additionally, we observed an increased sensitivity to prexasertib in the presence of MNA, which is a genetically distinct subtype of NB that is associated with poor patient outcome (23–25). While 11q loss and MNA are very different from one another on a genomic level, literature suggests that cells with these aberrations have one thing in common: replication stress. Previous studies have shown that 11q loss induces in haploinsufficiency of key DNA damage repair genes, which results in the accumulation of DNA damage and thus, replication stress (34). Independently, it has been shown that MNA is also capable of inducing the accumulation replication stress by activating dormant origins of replication (27, 35). Despite being very different genomic aberrations, these studies have shown that increased replication stress associated with 11q loss or MNA induces increased sensitivity to inhibition of key replication stress proteins (27, 34–38). As CHK1 is essential to the replication stress response *via* its role in replication fork stabilization and modulation of the S-phase and G_2_-M cell cycle checkpoints during DNA damage repair, it is perhaps unsurprising that we observe effective cell killing when CHK1 is inhibited in cells with genomic aberrations that are known to cause additional replication stress. The dependency of NB cells on replication stress response pathways for survival is further elucidated in a recently published study where it is demonstrated that MNA-driven NB is resistant to replication stress *via* overexpression of ribonucleotide reductase subunit M2 (RRM2) (35).

Investigation into compounds that act synergistically with prexasertib further support our hypothesis that CHK1 sensitivity is related to inherent replication stress driven by genomic aberrations in NB. In our study, the most synergistic combination observed was when prexasertib was combined with adavosertib, a WEE1 inhibitor. WEE1 kinase regulates cell cycle progression by inhibiting mitotic progression at the G_2_ checkpoint. Considering that CHK1 also plays an important role in cell cycle progression, it is not unexpected that abrogation of both CHK1 and WEE1 signaling pathways would be exceptionally lethal as cells with DNA damage would inexorably be forced into mitosis. In fact, the potency of combined CHK1 and WEE1 inhibition has been explored in NB and a mechanism governed by DNA damage accumulation and mitotic catastrophe has been elucidated (39). Taking our monotherapy results into account, we further hypothesized that with the inherent dependency on cell cycle checkpoints induced by 11q loss or MNA, these cell lines would be the most sensitive to combined CHK1 and WEE1 inhibition. Consistently, combination therapy in 11q deleted or *MYCN* amplified cells resulted in strong synergy and improved *in vitro* efficacy; however, cells with a normal 11q locus and wild type *MYCN* also demonstrated strong synergism. A next step would be to further investigate the molecular consequences of 11q loss and test the *in vivo* effects of combined CHK1 and WEE1 inhibition in this context. Regardless of the mechanism, our results suggest that 11q loss and MNA—two distinct patient groups which encompass 70-80% of high-risk NB—might create a dependency on cell cycle checkpoints for survival, leading to a targetable vulnerability which can be exploited for therapeutic purposes (40–42).

In addition to combined targeted inhibitors, we also observed additive to minor synergistic effects when prexasertib was combined with the topoisomerase inhibitors topotecan and irinotecan. Using CHK1 as a chemotherapeutic potentiator is not entirely novel, however, it is interesting in the context of the standard-of-care treatment protocols for NB patients with relapsed or refractory disease (43). As per the SIOP-European Neuroblastoma (SIOPEN) and Children’s Oncology Group (COG) studies, topotecan and irinotecan have been introduced into the treatment protocols for patients with relapsed or refractory NB (44, 45). Furthermore, a recent study has presented combined CHK1 and topoisomerase inhibition as an effective combination in *KRAS* mutant colon cancer (46). Aberrations within the RAS-MAPK pathway are well-known in relapsed NB and finding the topoisomerase inhibitors topotecan and irinotecan as synergistic candidates in our study suggest that adding CHK1 inhibition to existing treatment protocols could potentially be beneficial for relapsed or refractory NB patients (47). To develop better therapeutic options for these patients, we believe this combination should also be investigated further.

Altogether our study highlights how we can use characteristic, large genomic aberrations to guide the development of novel drug targets and increase the potential applicability of these inhibitors in the clinic. Additionally, our data builds on the existing evidence that CHK1 is an effective therapeutic target for the treatment of NB that should be investigated further.

## Data availability statement

The datasets presented in this study can be found in online repositories. This data can be found here: https://www.ebi.ac.uk/ena/browser/home, accession number PRJEB54725.

## Author contributions

Conceptualization: KK, MD, and SH. Methodology: KK, TE, MB, SE, GT, MD, and SH. Investigation: KK, TE, LS, KH, VA-A, KO, and BK. Supervision: MB, JM, MD, and SH. Writing – original draft: KK. Writing – review and editing: TE, LL, GT, BY, JM, MD, SH. All authors contributed to the article and approved the submitted version.

## Funding

This project was financially supported by COMPASS consortium (Award No. ERAPERMED2018-121 within the ERAPerMED framework).

## Conflict of interest

The authors declare that the research was conducted in the absence of any commercial or financial relationships that could be construed as a potential conflict of interest.

## Publisher’s note

All claims expressed in this article are solely those of the authors and do not necessarily represent those of their affiliated organizations, or those of the publisher, the editors and the reviewers. Any product that may be evaluated in this article, or claim that may be made by its manufacturer, is not guaranteed or endorsed by the publisher.
