# Supplementary material for: Chromosome 11q loss and MYCN amplification demonstrate synthetic lethality with checkpoint kinase 1 inhibition in neuroblastoma
Source: Front Oncol. 2022 Sep 27;12:929123. doi: 10.3389/fonc.2022.929123 (PMC9552537; doi:10.3389/fonc.2022.929123)
Supplement: Supplementary file 2 [file DataSheet_2.docx]

Supplementary Material

# Supplementary Figures

**Supplementary Figure 1 |** Log fold change in area under the curve (AUC) for 11q deleted and wild type SKNSH clones following 72-hour incubation with compounds targeting the proteins involved in the pathways listed on the y-axis. Each dot represents a compound and prexasertib (CHK1 inhibitor), is highlighted in red.

**Supplementary Figure 2 |** Dose-response curves for classical NB cell lines following 72-hour exposure to prexasertib combined with adavosertib.

**Supplementary Figure 3 |** Heatmaps of bliss independence scores for each cell line across entire prexasertib and adavosertib dose-response matrix.

**Supplementary Figure 4|** Dose-response curves for classical NB cell lines following 72-hour exposure to prexasertib combined with SN-38.

**Supplementary Figure 5 |** Dose-response curves for classical NB cell lines following 72-hour exposure to prexasertib combined with topotecan.

**Supplementary Figure 6 |** Bliss independence heat maps for classical NB cell lines following 72-hour exposure to prexasertib combined with topotecan.

**(B)**

**(A)**

**Supplementary Figure 7 |** Dose-response curves for **(A)** 11q wild type NB tumoroids and **(B)** 11q deleted NB tumoroids following 72-hour exposure to prexasertib combined with adavosertib.


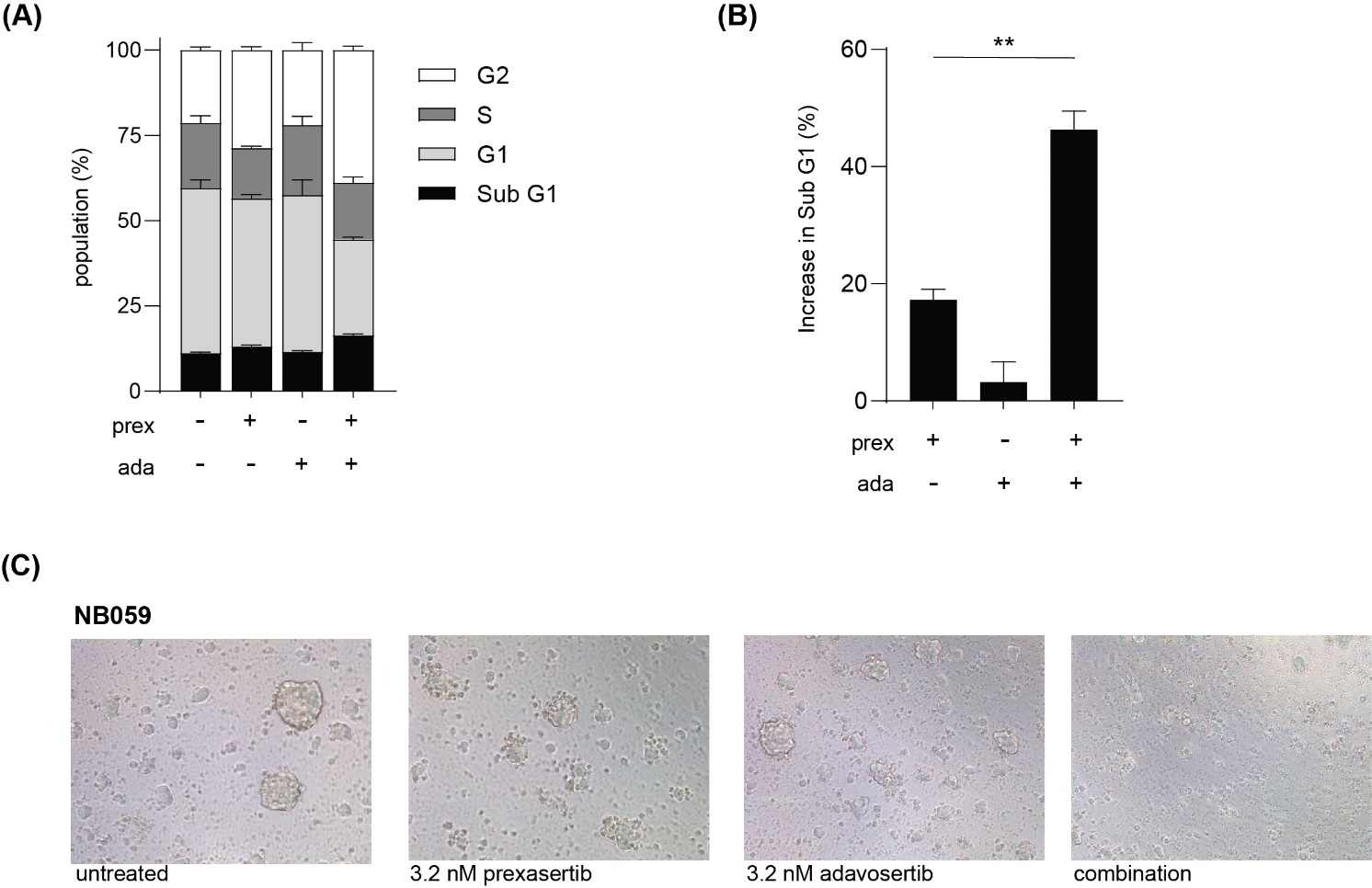


**Supplementary Figure 8** | **(A)** Cell cycle distribution of AMC772 cells following 72-hour treatment with 0.64 nM of prexasertib and/or 16 nM of adavosertib (n= 3). **(B)** Sub G1 fraction of AMC772 cells represented as percent increase relative to untreated control cells (n= 3) following 72-hour mono- or combination therapy with prexasertib and/or adavosertib. Statistical significance is reached (student’s t-test, p = 0.0014). **(C)** Phase contrast images of NB059 tumoroids following 72-hour incubation with prexasertib and/or adavosertib.
